# The seed‐specific transcription factor DPBF2 modulates the fatty acid composition in seeds

**DOI:** 10.1002/pld3.395

**Published:** 2022-04-03

**Authors:** Inyoung Kim, Kyeong‐Ryeol Lee, Mid‐Eum Park, Hyun Uk Kim

**Affiliations:** ^1^ Department of Molecular Biology Sejong University Seoul Republic of Korea; ^2^ Department of Agricultural Biotechnology, National Institute of Agricultural Sciences Rural Development Administration Jeonju Republic of Korea; ^3^ Department of Bioindustry and Bioresource Engineering, Plant Engineering Research Institute Sejong University Seoul Republic of Korea

**Keywords:** bZIP67, DPBF2, FAD3, FAE1, PDCT, seed fatty acid composition, transcription factor

## Abstract

Triacylglycerol (TAG), an ester derived from glycerol and three fatty acids (FAs), is synthesized during seed development and controlled by transcriptional regulation. We examined the mechanism regulating the FA composition of developing *Arabidopsis thaliana* seeds. The seed‐specific *DC3 PROMOTER‐BINDING FACTOR2* (*DPBF2*) transcription factor was upregulated by *LEAFY COTYLEDON2* (*LEC2*). DPBF2 showed transcriptional activity in yeast and localized to the nucleus in Arabidopsis protoplast cells. The Arabidopsis *dpbf2‐1* homozygous T‐DNA mutant and transgenic lines overexpressing of *DPBF*2 using a seed‐specific phaseolin promoter in wild‐type (WT) Arabidopsis and in *dpbf2‐1* showed similar FA composition profiles in their seeds. Their 18:2 and 20:1 FA contents were higher, but 18:1 and 18:3 contents were lower than that of WT. Transcript levels of *FATTY ACID DESATURASE2* (*FAD2*), *FAD3*, *LYSOPHOSPHATIDYLCHOLINE ACYLTRANSFERASE1* (*LPCAT1*), *LPCAT2*, *PHOSPHATIDYLCHOLINE DIACYLGLYCEROL CHOLINEPHOSPHOTRANSFERASE* (*PDCT*), and *FATTY ACID ELONGASE 1* (*FAE1*) are increased in *DPBF2*‐overexpressing seeds. Besides, *PDCT* and *FAE1* were upregulated by DPBF2, LEC1‐LIKE (L1L), and NUCLEAR FACTOR‐YC2 (NF‐YC2) transcriptional complex based on tobacco protoplast transcriptional activation assay. These results suggest that DPBF2 effectively modulates the expression of genes encoding FA desaturases, elongase, and acyl‐editing enzymes for modifying the unsaturated FA composition in seeds.

## 1 INTRODUCTION

Triacylglycerol (TAG) is an oil molecule composed of three fatty acid (FA) chains esterified to a glycerol backbone. TAG accumulates during seed development and is an energy source for seed germination and seedling establishment (Graham, 2008; Li‐Beisson et al., 2013). TAG and FA biosynthesis have been studied at the molecular level using *Arabidopsis thaliana* as a model oilseed (Ohlrogge et al., 1991; Somerville, 1991; Wallis & Browse, 2010).

Biosynthesis of seed FAs begins in plastids. The first step is the conversion of acetyl‐CoA to malonyl‐CoA by acetyl‐CoA carboxylase followed by the conversion of malonyl‐CoA to malonyl‐acyl carrier protein (ACP) through malonyl‐CoA‐ACP transacylase. Acetyl‐CoA and malonyl‐ACP are then condensed by 3‐ketoacyl‐ACP synthase III (KASIII) and elongated by two reductases and one dehydratase. Condensation by KASI is followed by subsequent elongation reactions to yield 16:0‐ACP. Condensation from 16:0‐ACP to 18:0‐ACP occurs via KASII. Plastidial desaturase then converts 18:0‐ACP to 18:1‐ACP which is transported as 18:1 to the cytosol by fatty acyl‐ACP thioesterase A (FATA); 16:0‐ACP and 18:0‐ACP are released as 16:0 and 18:0, respectively, to the cytosol by FATB. The resulting FAs form an acyl‐CoA pool.

The 18:1‐CoA are elongated to 20:1‐CoA and 22:1‐CoA by fatty acid elongase 1 (FAE1) and which contribute to the acyl‐CoA pool. Fatty acid desaturase 2 (FAD2) acts on 18:1 on the *sn‐2* position of phosphatidylcholine (PC) to create 18:2, which is converted to 18:3 by FAD3. The 18:2 and 18:3 (polyunsaturated fatty acid [PUFA]) are then released into the acyl‐CoA pool by acyl‐editing enzymes such as lysophosphatidylcholine acyltransferase (LPCAT). In addition, the PUFA‐PC is converted to PUFA‐diacylglycerol (DAG) via head group exchange by phosphatidylcholine diacylglycerol cholinephosphotransferase (PDCT) (Bates et al., 2012). The PUFA‐enriched acyl‐CoA pool and PUFA‐DAG are used for TAG biosynthesis in oilseeds (Bates & Browse, 2012).

TAG biosynthesis occurs in the endoplasmic reticulum (ER), usually via the Kennedy pathway. The first FA is attached to glycerol 3‐phosphate (G3P) at its *sn‐1* position by glycerol‐3‐phosphate acyltransferase (GPAT), yielding lysophosphatidic acid (LPA). The second FA is attached to the *sn‐2* position of LPA by lysophosphatidic acid acyltransferase (LPAT), resulting in phosphatidic acid (PA). PA phosphatase (PAP) removes the phosphate at the *sn‐3* of PA to produce DAG. Finally, the third FA is attached to the *sn‐3* position by diacylglycerol acyltransferase (DGAT) to produce TAG. Kennedy pathway utilizes FAs in the acyl‐CoA pool and is thus acyl‐CoA dependent. TAG can also be produced without incorporating FAs from the acyl‐CoA pool, in which phospholipid:DAG acyltransferase (PDAT) transfers the FA at the *sn‐2* position of PC to the *sn‐3* position of DAG (Dahlqvist et al., 2000; Ståhl et al., 2004). TAG is concentrated between the ER bilayer and surrounded by the hydrophobic protein oleosin, which accumulates in the embryo cell body in the form of a spherical oil body (Lacey et al., 1999; Napier et al., 1996; Wanner & Theimer, 1978).

Although the genes encoding enzymes involved in FA biosynthesis are well known, there are insufficient studies on the transcription factors that regulate the contents of TAG and unsaturated FAs during seed development. The master regulators LEAFY COTYLEDON1 (LEC1), ABSCISIC ACID INSENSITIVE3 (ABI3), FUSCA3 (FUS3), and LEC2 regulate seed development and TAG biosynthesis (Giraudat et al., 1992; Keith et al., 1994; Meinke, 1992; Stone et al., 2001; West et al., 1994). Mutations in these master regulator genes alter the FA composition and/or decrease the TAG content of seeds, whereas ectopic overexpression of master regulators promotes FA biosynthesis and TAG accumulation (Kim et al., 2014; Lemieux et al., 1990; Mu et al., 2008; Santos‐Mendoza et al., 2005). WRINKLED1 (WRI1) is a transcription factor regulating genes involved in glycolysis and FA biosynthesis (Baud et al., 2007; Cernac & Benning, 2004; Focks & Benning, 1998). LEC1, LEC2, and FUS3 bind to the promoter of *WRI1*, regulating its expression (Baud et al., 2007; Kong et al., 2019; Marchive et al., 2014; Mu et al., 2008).

Expression of *LEC2* under the control of a senescence‐specific promoter results in TAG accumulation in leaves (Kim et al., 2014, 2015). *LEC2* expression in leaves induces various seed‐specific transcription factor genes, presumed to be downstream targets of LEC2 (Kim et al., 2015). Among these seed‐specific transcription factors, we examined those that were coexpressed with FA and TAG biosynthesis genes. *DC3* promoter‐binding factor 2 (DPBF2) is strongly upregulated in *LEC2*‐expressing leaf tissue and is coexpressed with FA biosynthesis genes, suggesting that DPBF2 is a transcription factor controlling FA biosynthesis (Kim et al., 2015). DPBF2 is also known as BASIC LEUCINE ZIPPER TRANSCRIPTION FACTOR 67 (bZIP67) (Jakoby et al., 2002). Genes encoding these DPBF proteins were initially isolated from a sunflower (*Helianthus annuus*) immature seed library using a modified yeast one‐hybrid system (Kim et al., 1997; Kim & Thomas, 1998). Of five *DPBF* genes expressed in Arabidopsis seeds, DPBF2 was confirmed to bind to the *DC3* promoter in an electrophoretic mobility shift assay (EMSA) and showed transcriptional activity in a yeast one‐hybrid system (Kim et al., 2002).

DPBF2/bZIP67 has already been reported to be a transcription factor regulating *FAD3* (Mendes et al., 2013). In this study, we aim to confirm that DPBF2 is a seed‐specific transcription factor and regulated transcriptionally by LEC2. We also analyzed the FA composition and FA‐related genes in a *dpbf2‐1* knock‐out mutant and transgenic lines overexpressing *DPBF2* in seeds. We confirmed that DPBF2 upregulates the expression of *PDCT* and *FAE1* together with LEC1‐LIKE (L1L) and NUCLEAR FACTOR‐YC2 (NF‐YC2) by transcriptional activation assay in the tobacco protoplast.

## 2 MATERIALS AND METHODS

### 2.1 Plant materials and growth conditions

Plants including wild‐type (WT) *A. thaliana* (ecotype Col‐0), the T‐DNA insertion mutants, *dpbf2‐1* (Salk_085497C) and *lec2‐1* (CS3868) (Gaj et al., 2005; Meinke et al., 1994), F2 generation plants generated by WT and *dpbf2‐1* crossing, and transgenic plants overexpressing *DPBF2* under the control of cauliflower mosaic virus (CaMV) 35S promoter or phaseolin seed‐specific promoter (Slightom et al., 1983) were grown in potting soil in a growth chamber at 22 °C under a 16 h light/8 h dark period.

### 2.2 Transcriptional activity assay in protoplast and yeast

To perform the transcriptional activity assay in protoplast, recombinant effector and reporter plasmids were cotransformed into tobacco protoplasts by polyethylene glycol‐mediated transformation (Yoo et al., 2007). The effector plasmids were constructed with CaMV 35S promoter fused to *LEC2*, *DPBF2*, *L1L*, and *NF‐YC2* cDNA genes, whereas the reporter plasmids were constructed with *DPBF2*, *FAD2*, *LPCAT1/2*, *PDCT*, *FAE1*, and *CRU3* promoter, respectively, including 5′‐untranslated region (UTR) fused to luciferase reporter gene using listed primers (Table S1). To generate mutated *DPBF2* promoter sequence, two primers including “AAAAAAAAAA” were designed, and it was amplified by two times PCR. GUS activity using pBI221 vector measured for normalization of the luciferase activity. These effector and reporter plasmids were cotransfected using polyethyleneglycol (PEG) solution (40% PEG 4000, 200 mM mannitol, 100 mM CaCl_2_) to protoplast isolated from *Nicotiana benthamiana* using enzyme solution (400 mM mannitol, 20 mM KCl, 20 mM MES [pH 5.7], 0.25% macerozyme, 1% cellulase). And this protoplast was incubated in dark for 16 h and measured luciferase and GUS activity with a luminometer (Glomax 20/20; Promega, USA).

The transcriptional activity of DPBF2 (AT3G44460) was assayed in budding yeast (*Saccharomyces cerevisiae*). A *DPBF2* 5′‐end primer containing a *Bam*HI site (5′‐GCGGATCCGTTCGGTTTTCGAATCGGAGAC‐3′) and 3′‐end primer containing a *Pst*I site (5′‐GGGCTGCAGTTACCACCCGGCACTGGCC‐3′) were used to PCR‐amplify *DPBF2* cDNA. The *DPBF2* cDNA was digested with *Bam*HI and *Pst*I and cloned into a pGBKT plasmid vector containing a GAL4 DNA‐binding (DB) domain to produce the pGBKT‐DB‐DPBF2 vector capable of expressing the *DB‐DPBF2* fusion gene product. This vector was transformed into yeast strain PBN204, harboring *ADE2* and *URA3* reporter genes that were expressed under the control of various *GAL* promoters. pACT2 containing the GAL4 transcriptional activation domain was used as a positive control. Yeast transformants were selected on SD‐LW plates containing SD minimal media without leucine (L) or tryptophan (W). The selected colony was replicated to determine transcriptional activity on SD‐LWU medium without leucine, tryptophan, and uracil (U) and on SD‐LWA without leucine, tryptophan, and adenine (A).

### 2.3 Gene cloning and vector construction

To construct a plant transformation vector capable of overexpressing *DPBF2*, *DPBF2* cDNA was amplified by RT‐PCR from RNA isolated from developing Arabidopsis siliques and cloned into the pENTR‐D/TOPO vector (Invitrogen, USA). The nucleotide sequence of the pENTR‐*DPBF2* cDNA clone was determined by Sanger sequencing. Plant recombinant expression vectors *35S‐DPBF2* and *Ph‐DPBF2*, in which *DPBF2* is overexpressed under the control of the CaMV 35S promoter and a seed‐specific phaseolin promoter, respectively, were generated by LR clonase cloning in pEarleyGate201 (11.7 kb) and pPhaseolin‐Gate plant expression vectors (Kim et al., 2020) (see Figures S3A and 6a). The *Ph‐GUS* transformation control vector was generated using the LR clonase cloning reaction between pENTR‐GUS and pPhaseolin‐Gate (Figure 6a). *Agrobacterium tumefaciens* GV3101 was transformed with the plant expression vectors, and the Agrobacterium‐mediated Arabidopsis transformation was carried out by the floral‐dip method (Clough & Bent, 1998). Transgenic Arabidopsis plants were selected on MS medium containing 50 μg ml^−1^ kanamycin for *35S*‐*DPBF2* and by spraying with BASTA herbicide at a concentration of 0.3% (v/v) for *Ph*‐*DPBF2* and *Ph‐GUS*.

### 2.4 Subcellular localization

*DPBF2* cDNA was amplified by PCR from pENTR‐DPBF2 using primers with *Bam*HI and *Sac*I restriction sites and cloned into p326‐eGFP vector (Lee et al., 2016) carrying the enhanced green fluorescent protein (eGFP) to construct vector DPBF2‐GFP. This vector was transformed into Arabidopsis protoplasts using the PEG method with a red fluorescent protein (RFP) vector targeted at the nucleus as a control (Jin et al., 2001). Fluorescence images showing intracellular localization of gene products were obtained using a fluorescence microscope (Axioplan 2; Carl Zeiss, Germany).

### 2.5 T‐DNA mutant analysis

Salk_085497C seeds, with T‐DNA inserted into *DPBF2*, were purchased from ABRC (Arabidopsis Biological Resource Center). To obtain a homozygous *dpbf2‐1* T‐DNA insertion mutant, *DPBF2* gene‐specific primers targeting sequences either side of the T‐DNA insertion site (LP‐primer: 5′‐ACGATGTAATTTCAGCATCGG‐3′; RP‐primer: 5′‐CTCGGTTTTGGGAGAATCTTC‐3′) and the T‐DNA specific primer LBb1.3 (5′‐ATTTTGCCGATTTCGGAAC‐3′) were used for PCR. RT‐PCR was conducted using total RNA extracted from developing siliques of WT and *dpbf2‐1* mutants using primers targeting the 5′‐end (5′‐ATGTCGGTTTTCGAATCGGAG‐3′) and 3′‐end (5′‐TTACCACCCGGCACTGGCCAT‐3′) to cover the full length of transcripts (see Figure S1A). *ACT2* (At3g18780) forward (5′‐ATGATGCTCCCAGGGCTGTTT‐3′) and reverse (5′‐TTGTCACACACAAGTGCATCA‐3′) primers were used for control RT‐PCR amplification. Primer sequences for *DPBF2* expression analysis in developing siliques of WT and *dpbf2‐1* by reverse‐transcription quantitative PCR (RT‐qPCR) analysis were designed based on nucleotide sequences both 5′ and 3′ with the T‐DNA insertion centered in the *DPBF2* genome. The forward primer sequence is 5′‐TTGATGGAGCGGAGACAACG‐3′ at the end of first exon of *DPBF2* genomic DNA, and the reverse primer sequence is 5′‐CACTGGCCATCCTCCGAATC‐3′ at the end of fourth exon (Figure S1A). The DPBF2 cDNA size amplified by RT‐qPCR is 250 base pair.

### 2.6 Reverse‐transcription PCR and quantitative PCR

Total RNAs were isolated from S6 stage siliques containing walking‐stick embryo using the previously reported method (Onate‐Sanchez & Vicente‐Carbajosa, 2008) and from other tissues using TRIzol reagent (Invitrogen, USA). Quantitative PCR was performed with gene‐specific primers and cDNA prepared from 1 μg total RNA from developing siliques of WT, *dpbf2‐1*, *35S‐DPBF2*, *Ph‐DPBF2*, and *Ph‐GUS* plants. Quantitative PCR was performed using SYBR Green Premix Ex Taq II (Takara, Japan) and the CFX96 Real‐Time PCR system (Bio‐Rad Laboratories), as specified by the manufacturer. Housekeeping genes *ACTIN2* and *elF4a* were used for normalization in RT‐qPCR analysis. Primers for RT‐qPCR are listed in Table S1.

### 2.7 Fatty acid analysis

Ten milligrams of dry seeds and two leaf samples were transmethylated at 85°C for 90 min in 0.5 ml toluene and 0.5 ml 5% (v/v) H_2_SO_4_ in methanol. Heptadecanoic acid (17:0) was added to each sample as an internal standard to measure the amount of FAs. After transmethylation, 1.5 ml 0.9% (w/v) NaCl solution was added, and fatty acid methyl esters (FAMEs) were extracted with 0.5 ml *n*‐hexane. FAMEs were analyzed using gas chromatography (GC) on a GC‐2010 Plus Gas Chromatograph (Shimadzu, Japan) with a 30 m × 0.25 mm (inner diameter) HP‐FFAP column (Agilent, USA) while the oven temperature was increased from 190°C to 230°C at 3°C min^−1^. Nitrogen was used as the carrier gas at a flow rate of 1.4 ml min^−1^.

### 2.8 Data repetition and statistical analysis

Expression analysis of *LEC2* and *DPBF2* was performed using the results of three repeated microarray analyses from two independent transgenic lines, OIL21 and OIL25, which express *LEC2* under the control of senescence‐inducible promoter (Kim et al., 2015). The subcellular localization experiments using *DPBF2‐GFP* construct were performed twice. Seed fatty acid analysis of WT and *dpbf2‐1* T‐DNA mutants was performed in 14 independent lines. Fatty acid composition of *35S‐DPBF2* overexpressing transgenic line seeds was analyzed in nine T1 independent lines. Seed fatty acid composition of WT + *Ph‐GUS*, WT + *Ph‐DPBF2*, and *dpbf2‐1* + *Ph‐DPBF2* was analyzed from T3 seeds obtained from three T2 independent lines. RT‐qPCR analysis for genes between WT and *dpbf2‐1* mutant and between WT + *Ph‐GUS* and WT + *Ph‐DPBF2* transformed plants was analyzed from three independent samples. Each data represents the mean (±SE) from three independent biological replicates. Asterisks indicate significant changes compared with control (*p < .05, **p < .01, ***p < .001).

## 3 RESULTS

### 3.1 DPBF2 is upregulated by LEC2

**FIGURE 1:**
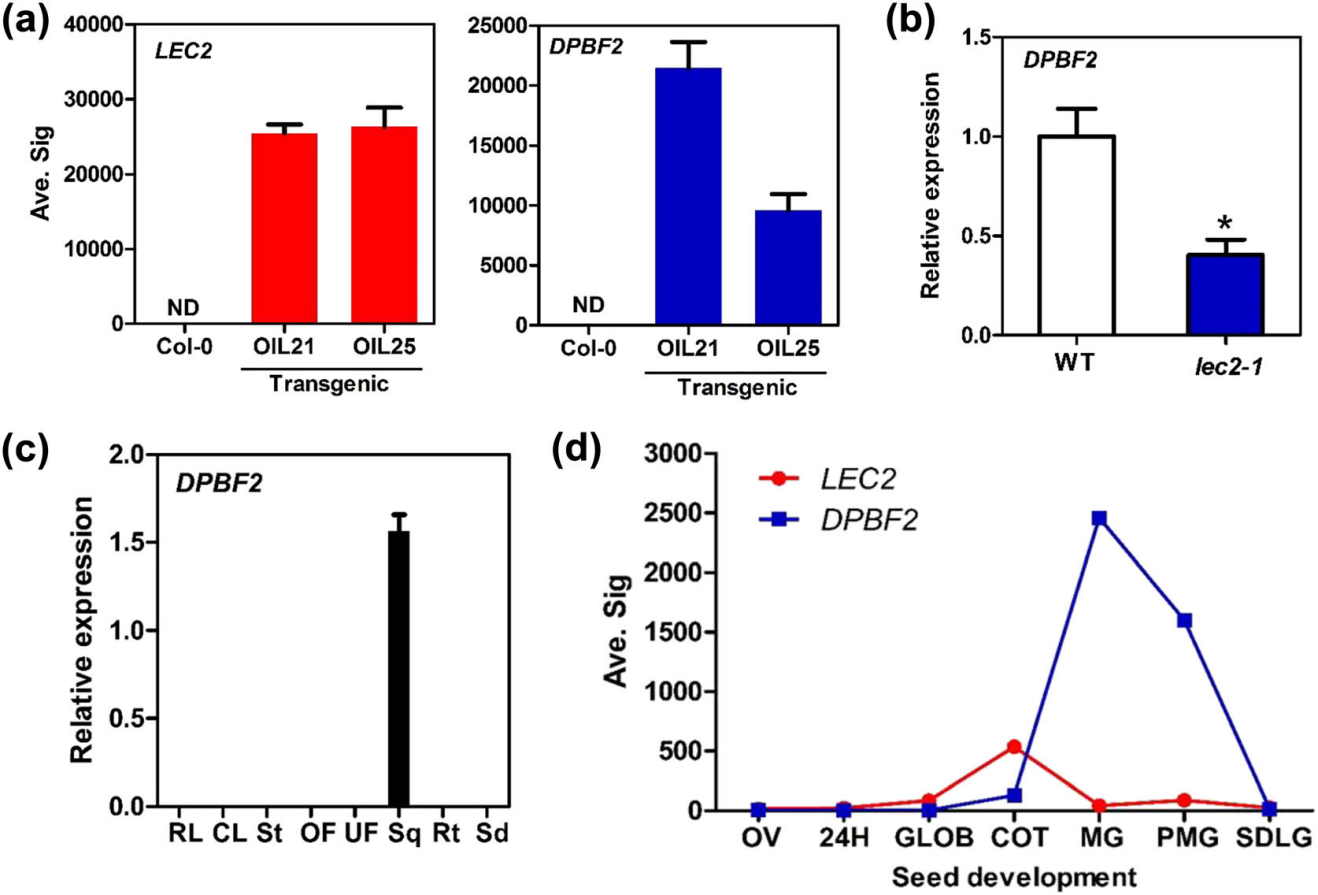
*DPBF2* expression in transgenic lines OIL21 and OIL25, harboring senescence‐inducible *LEC2*, and wild‐type (WT) Arabidopsis plants. (a) Senescence‐inducible *LEC2* upregulates *DPBF2* transcript levels in senescing leaves. Senescent leaves of WT and two independent transgenic lines, OIL21 and OIL25, were used for microarray analysis. Relative expression values are mean (±SE) from three independent biological replicates. ND: none detected. (b) *DPBF2* expression in developing siliques of the *lec2‐1* mutant. Relative expression values are given in comparison with the WT (WT = 1). Mean (±SE) values are obtained from three independent biological replicates. *p < .05 (unpaired *t* test). (c) Seed‐specific expression of *DPBF2*. RL: rosette leaf; CL: cauline leaf; St: stem; OF: open flower; UF: unopen flower; Sq: developing silique S6 stage (containing walking‐stick embryo stages); Rt: root; Sd: seedling. Relative expression values are represented with a mean (±SE) from three independent biological replicates. (d) *LEC2* and DPBF2 expression before, during, and after seed development. Average signal intensity (Avg. Sig.) of transcripts detected by GeneChip (Le et al., 2010). OV: embryo sac; 24H: pre‐globular embryo; GLOB: globular embryo; COT: linear and bent embryo; MG: mature embryo; PMG: post mature embryo; SDLG: seedling

To examine if DPBF2 is regulated by LEC2, we used the Arabidopsis OIL21 and OIL25 transgenic lines in which LEC2 is expressed under the control of a senescence‐inducible promoter, resulting in TAG biosynthesis and accumulation in the leaf tissue (Kim et al., 2015). We found that DPBF2 was strongly upregulated in the senescing leaves of the transgenic plants compared with those of the WT (Figure 1a). Besides, DPBF2 expression was significantly lower in the developing seeds of *lec2‐1* mutant than WT seeds (Figure 1b). These results suggest that DPBF2 may be regulated directly or indirectly by LEC2. Our results are also consistent with the microarray analysis of senescing leaves performed by Kim et al. (2015) which reveals 45 seed‐specific transcription factors including DPBF2 that were upregulated in the leaves of OIL21 compared with those of WT Arabidopsis (Table S2).

To analyze the expression of *DPBF2*, we performed RT‐qPCR using a *DPBF2* gene‐specific primer set and total RNA samples from several tissues of WT Arabidopsis (Table S1). *DPBF2* transcripts were detected only in developing seeds, confirming that *DPBF2* is a seed‐specific gene (Figure 1c). We investigated the expression pattern of *DPBF2* before, during, and after seed development using GeneChip microarray data from Le et al. (2010). *LEC2* expression was detected from the globular stage and peaked during cotyledon development (linear and bent stage of embryo). *DPBF2* transcripts were detected later than those of *LEC2* and peaked at the mature embryo stage when TAG had accumulated (Figure 1d).

**FIGURE 2:**
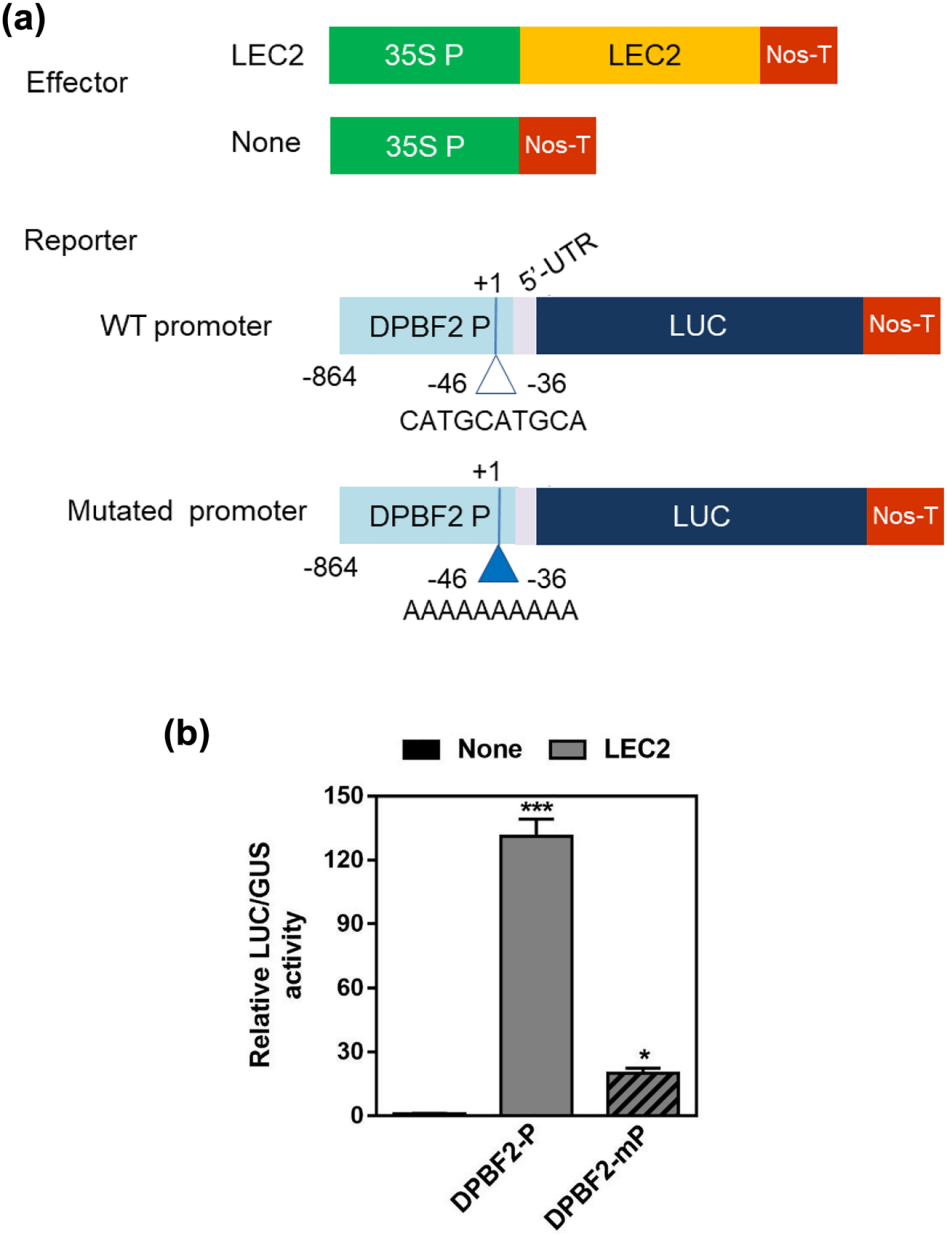
*DPBF2* transcriptional activation assay by LEC2 in *Nicotiana benthamiana* leaf protoplasts. (a) Schematic diagrams of reporter and effector constructs. In the effector construct, LEC2 was cloned between the CaMV 35S promoter and the terminator of the nopaline synthase gene (Nos‐T). In the reporter constructs, the WT and RY motif‐mutated promoter of *DPBF2* were fused to the luciferase gene. (b) WT and DPBF2 promoter activation assay in *Nicotiana benthamiana* leaf protoplasts. The effector and reporter constructs shown in (a) were cotransfected into protoplasts, and luciferase activities were determined fluorometrically. *GUS* gene expression was used to normalize the luciferase activities, and five measurements were averaged (*t* test, *p < .05). The bars indicate the SEM

To confirm that LEC2 regulates *DPBF2* transcriptional activity by binding to its promoter, we investigated the luciferase activity driven by the promoter of 1092 bp including the 5′‐UTR portion of *DPBF2* in transformed *Nicotiana benthamiana* protoplast (Figure 2). As a result, when LEC2 was expressed as an effector, *DPBF2* promoter activity was increased by 130 times compared with the controls without effector expression. However, when all 10 base pairs of the RY motif (CATGCATGCA) predicted as the binding site of LEC2 (Braybrook et al., 2006) were replaced with A, the activity was greatly reduced by six to seven times compared with the native promoter (Figure 2b). This result suggests the possibility that LEC2 directly binds to the RY motif present in the −36 to −46 region of the *DBPF2* promoter, resulting in regulating the transactivation of *DPBF2* during seed development. Taken all together, these results showed that LEC2 directly regulates the expression of *DPBF2*.

### 3.2 DPBF2 has transcriptional regulatory activity and is present in the nucleus

**FIGURE 3:**
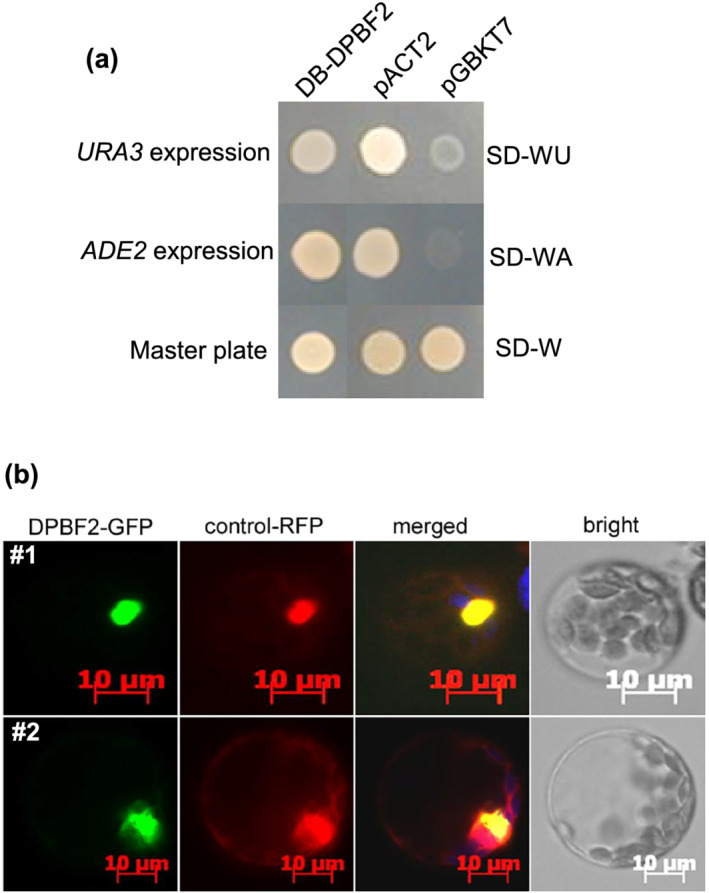
Transcriptional activity and subcellular localization of DPBF2. (a) Transcription activity test of DPBF2 in yeast. DB‐DPBF2, associated with the GAL4 DNA‐binding (DB) domain, induces *URA3* and *ADE2* expression in transgenic yeast, allowing growth in medium lacking uracil and adenosine. A positive control vector (pACT2) expressing the GAL4 activation domain (AD) allows the expression of *URA3* and *ADE2* in yeast. Negative control yeast expressing the pGBKT7 vector lacking the GAL4 DB domain and the GAL4 AD did not grow because *URA3* and *ADE2* were not expressed. SD‐W: minimum medium without tryptophan; SD‐WA: minimum medium without tryptophan and adenosine; SD‐WU: minimum medium without tryptophan and uracil. (b) DPBF2 localizes in the nucleus of Arabidopsis protoplasts. A nuclear‐targeted RFP construct (control‐RFP) was used as a control. Two independent experiments (#1 and #2) showed the same results

To further investigate the function of DPBF2 as a transcription factor, *DPBF2* was fused to the GAL4 DB domain of yeast and transformed into yeast PBN204, a nutrient‐requiring strain. The *DB‐DPBF2*‐expressing yeast cells grew as normal as those harboring the positive control clone (pACT2) under culture conditions without adenine or uracil, demonstrating that DPBF2 has transcriptional activity. By contrast, the negative control yeast transformed with the pBGKT7 vector did not grow under the same conditions (Figure 3a). To investigate if DPBF2 is localized in the nucleus, we introduced a construct containing *GFP* fused to *DPBF2* together with a nuclei marker RFP vector into Arabidopsis protoplasts. In two independent experiments, the green fluorescence of DPBF2‐GFP was present in the nucleus and colocalized with the nuclear‐located red fluorescence of the marker RFP (Figure 3b). Thus, we have demonstrated that DPBF2 is targeted to the nucleus and has transcriptional activity. It is evident that DPBF2 is a functional transcription factor.

### 3.3 A *dpbf2‐1* T‐DNA insertion mutant lacks DPBF2 expression in developing seeds

To investigate the function of *DPBF2*, we identified homozygous Arabidopsis T‐DNA insertion mutants from seeds of Salk_085497C line (Figure S1). *DPBF2* contains four exons and three introns. We selected a mutant with the T‐DNA inserted into the second intron, which we named *dpbf2‐1* (Figure S1A). As expected, PCR analysis using *DPBF2* gene‐specific primers located on the left and right sides of the T‐DNA insertion position amplified a 1.2‐kb band in WT, whereas the LBb1.3 primers located within the T‐DNA yielded a 0.6‐kb band in the *dpbf2‐1* mutant (Figure S1B).

We further analyzed *DPBF2* expression by RT‐PCR of total RNA isolated from the developing seeds of WT and *dpbf2‐1* mutant plants using *DPBF2* specific primers that recognize cDNA containing the full‐length sequence of *DPBF2*. As shown in Figure S1C, the *DPBF2* transcript was detected in WT, but not in *dpbf2‐1*. Thus, although the T‐DNA is inserted into the second intron, splicing did not occur correctly, resulting in the absence of *DPBF2* expression. In addition, RT‐qPCR was performed using primers of the sequence corresponding to the exon portion of left and right regions of T‐DNA insertion to investigate whether the *DPBF2* transcript was expressed in the developing seed of the *dpbf2‐1* mutant. As a result, *DPBF2* transcript was not detected in the *dpbf2‐1* mutant (Figure S1D).

### 3.4 Fatty acid composition is changed in *dpbf2‐1* mutant seeds

**FIGURE 4:**
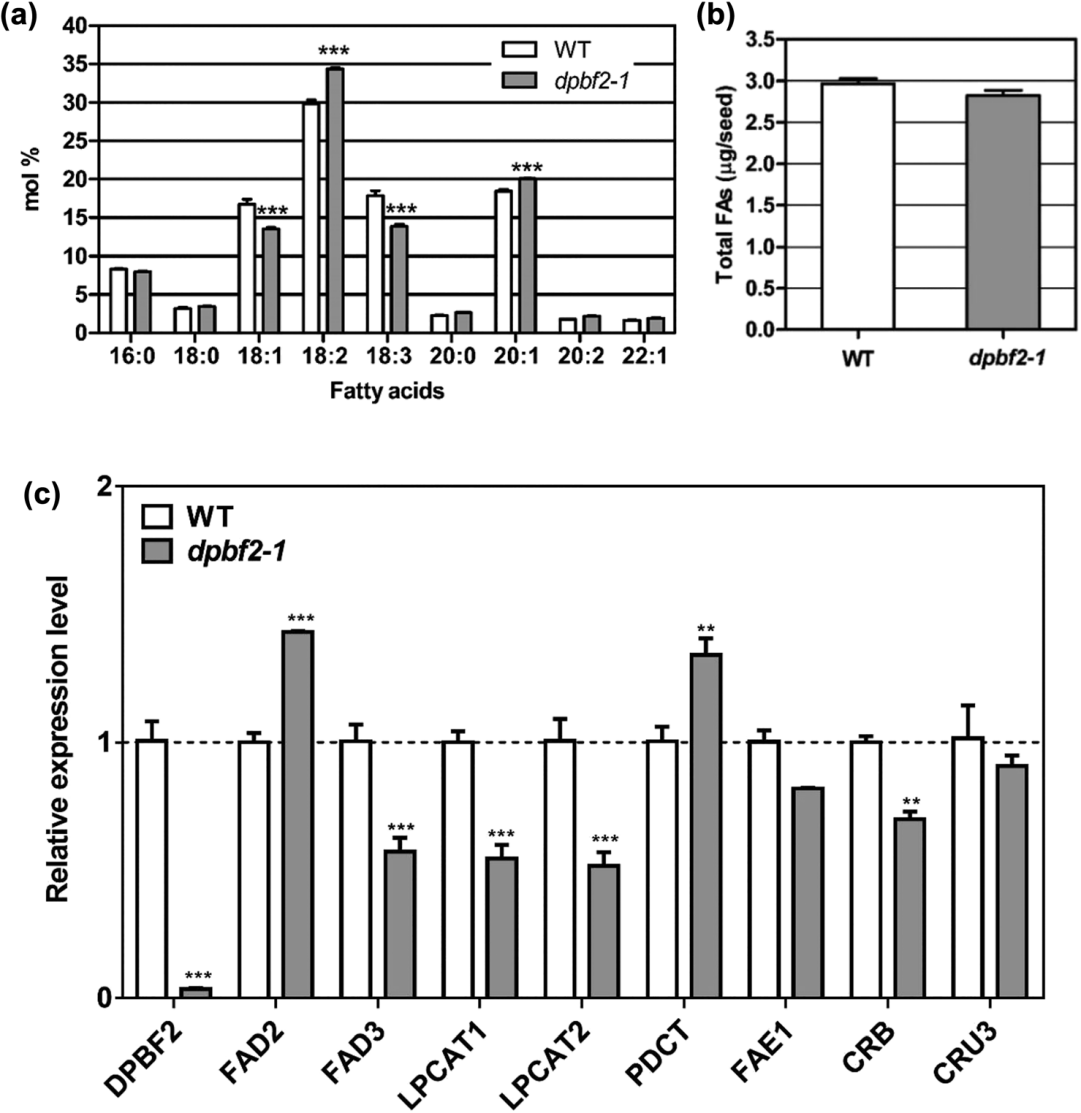
Seed fatty acid content analysis and gene expression changes in WT and *dpbf2‐1* knock‐out mutant. (a) Seed FA composition in WT and *dpbf2‐1* line. Statistically significant differences are indicated by two‐way ANOVA with Bonferroni posttests (***p < .001). (b) Total fatty acid amount in WT and *dpbf2‐1* line. A statistically significant test for total fatty acid amount was done by *t* test with Wilcoxon matched pairs test in 95% confidence intervals. Data represent the mean (±SE) from 10 independent biological replicates. (c) Gene expression changes in developing seeds of the WT and *dpbf2‐1* line by RT‐qPCR analysis of *DPBF2*, *FAD2*, *FAD3*, *LPCAT1/2*, *PDCT1*, *FAE1*, *CRB*, and *CRU3* transcript levels. The measurements are normalized to *eIF4a*. Statistically significant differences are indicated by one‐way ANOVA with Tukey *t* tests (**p < .01, ***p < .001)

To examine the effect of expressing *DPBF2* in Arabidopsis, we compared the seed FA composition of *dpbf2‐1* with that of the WT (Figure 4a). There was no difference in the ratio of 16:0 to 18:0 saturated FAs in *dpbf2‐1* seeds compared with WT seeds, but the contents of unsaturated FAs such as 18:1, 18:2, 18:3, and 20:1 were significantly changed at *p* < .001 by two‐way ANOVA. The 18:1 and 18:3 FAs showed a decrease (3% and 4%) in *dpbf2‐1* compared with the WT. The 18:2 and 20:1 showed an increase of 5 and 2%, respectively, in *dpbf2‐1* compared with the WT (Figure 4a). Total FA content representing the TAG content was not significantly different between the WT and mutant at *p* < .05 by *t* test (Figure 4B). There was also no difference in FA composition of leaves between the WT and *dpbf2‐1* mutant (Figure S2). Therefore, DPBF2 affected unsaturated FA composition in seed oil without change of its total TAG content. DPBF2 did not affect FA composition in leaves.

Because the unsaturated FA composition of seeds was altered relative to the WT in the *dpbf2‐1* mutant line (Figure 4a), the DPBF2 transcription factor may regulate the expression of genes involved in unsaturated FA biosynthesis in developing seeds. We thus compared the expression of genes involved in unsaturated FA biosynthesis and acyl‐editing pathway in the 13‐day‐old developing seeds after pollination of *dpbf2‐1* knock‐out mutant and the WT (Figure 4c). To determine the accuracy of RT‐qPCR analysis, *CRUCIFERIN B* (*CRB*) and *CRUCIFERIN 3* (*CRU3*) were used as controls among the seed storage protein genes that were previously reported to have decreased expression in the developmental seeds of *dpbf2‐1*/*bzip67* knock‐out mutant (Mendes et al., 2013). Gene expression analysis was performed using samples of developing WT and *dpbf2‐1* mutant seeds at the same stage. In developing seeds of the *dpbf2‐1* mutant that did not express *DPBF2*. Similar to a previous report (Mendes et al., 2013), the expression of *CRB* and *CRU3* was decreased compared with the WT. The expression of the unsaturated FA biosynthesis gene *FAD2* slightly increased and *FAD3* decreased. The decrease in *FAD3* expression in the *dpbf2‐1* mutant was consistent with results from previously reported *dpbf2‐1*/*bzip67* mutants (Mendes et al., 2013). The increase of *FAD2* expression and decrease of *FAD3* expression in the *dpbf2‐1* mutant was also consistent with the 18:2 FA increase and 18:1 and 18:3 FA decrease phenotype (Figure 4a). Among the genes encoding enzymes in the acyl‐editing pathway between PC and TAG, expression of *LPCAT1* and *LPCAT2* was lower in the *dpbf2‐1* mutant than in the WT, while *PDCT* expression was slightly increased (Figure 4c). The change of the unsaturated FA composition in *dpbf2‐1* developing seeds was obvious, but the change in the FA synthesis genes showed less than onefold.

### 3.5 *DPBF2/dpbf2‐1* heterozygous showed intermediate FA composition levels between WT and *dpbf2‐1* mutant in seeds

**FIGURE 5:**
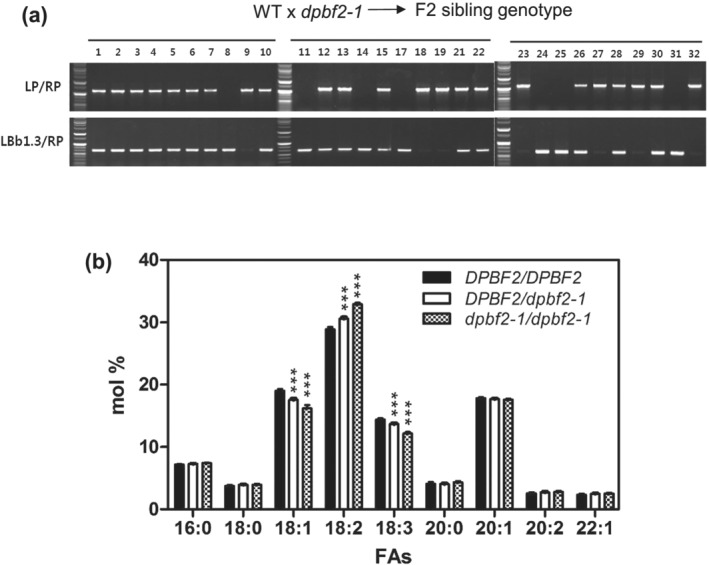
Progeny segregation test for *DPBF2* and its effect on FA composition. (a) The *dpbf2‐1* homozygous mutant was crossed with WT and a progeny F_1_ heterozygous plant was selfed to produce F_2_ lines. The F_2_ plant seeds were germinated, and 30 individual plants were genotyped using genomic DNA PCR to identify WT and heterozygous or homozygous *dpbf2‐1* T‐DNA genotypes. Genotyping of 30 F2 offspring resulted in 7 WT (*DPBF2/DPBF2*), 16 heterozygous (*DPBF2/dpbf2‐1*), and 7 homozygous (*dpbf2‐1/dpbf2‐1*). The *dpbf2‐1* T‐DNA insertion locus segregates as a single copy. (b) The fatty acid composition in F_2_ segregants of *DPBF2/dpbf2‐1* F1 heterozygous plants. Statistically significant differences from WT were determined by two‐way ANOVA with Bonferroni posttests (***p < .001)

To further elucidate the effect of *DPBF2* on seed FA composition, we crossed WT Arabidopsis with *dpbf2‐1* to create *DPBF2*/*dpbf2‐1* heterozygous lines in the F_1_ generation. F_2_ segregating progenies were generated by F_1_ selfing, and 30 F_2_ lines were randomly selected. We determined the *DPBF2* genotype of 30 F_2_ plants and analyzed their seed FAs (Figure 5). In an F_2_ generation of 30 individuals, null‐segregated WT lines, heterozygous lines, and homozygous lines segregated 7:16:7, respectively, close to a 1:2:1 ratio (Figure 5a). We compared the average FA compositions of seeds obtained from WT, heterozygous, and homozygous individuals. In *dpbf2‐1* homozygous seeds, 18:1 and 18:3 FAs were decreased and 18:2 FA was increased compared with those of the WT (Figure 5b). Intriguingly, the seed FA composition of the *DPBF2*/*dpbf2‐1* heterozygous genotype showed a FA composition precisely intermediate between that of WT and *dpbf2‐1* homozygous mutants (Figure 5b). These results show that DPBF2 has a dosage‐dependent effect on FA composition.

### 3.6 Seed‐specific DPBF2 overexpressor regulates the expression of seed unsaturated FA biosynthesis genes

Because the seed FA composition of the *dpbf2‐1* knock‐out mutant decreased in 18:1 and 18:3 FAs and increased in 18:2 and 20:1 FAs compared with the WT (Figure 4a), we investigated changes in seed FA when DPBF2 was overexpressed. First, the *35S‐DPBF2* vector containing *DPBF2* cDNA expressed under the control of the CaMV 35S promoter was transformed into WT Arabidopsis (Figure S3A). We randomly selected nine T1 transgenic lines showing resistance to kanamycin and analyzed the FAs of their T2 seeds in comparison with the WT line. In all *35S‐DPBF2* T2 transgenic lines, *DPBF2* transcripts level was higher than that of WT (Figure S3B). *35S‐DPBF2* transgenic lines showed changes in 18:1, 18:2, 18:3, and 20:1 compared with those of WT. When the FA composition of *35S‐DPBF2* was compared with the FA composition of WT, the decrease of 18:1 and increase of 20:1 were not consistent in all transgenic lines, but the decrease of 18:2 and increase of 18:3 was consistent (Figure S3C).

**FIGURE 6:**
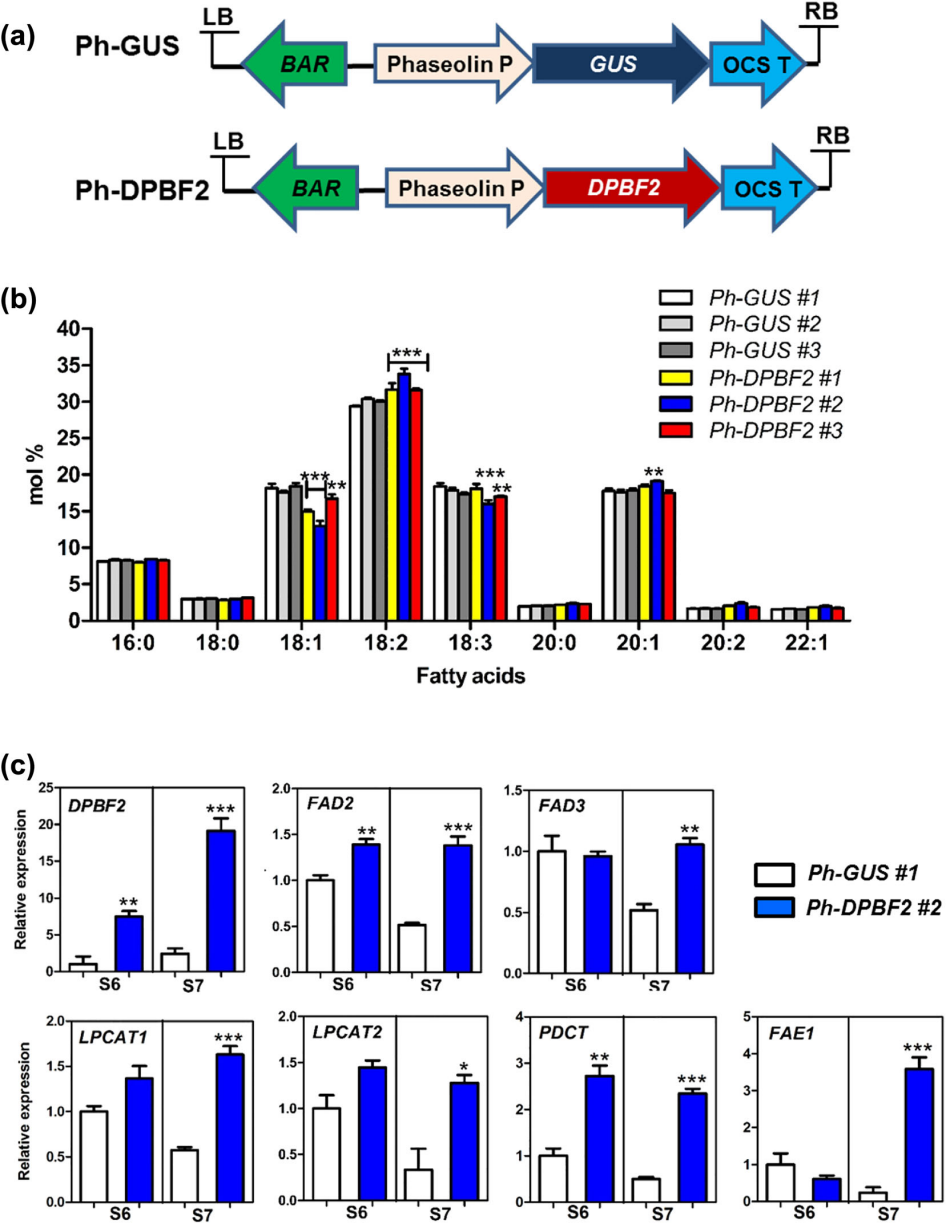
Fatty acid composition and gene expression changes in developing seeds of seed‐specific overexpressed *GUS* and *DPBF2* in WT background. (a) Vectors used for seed‐specific heterologous expression of *GUS* and seed‐specific overexpression of *DPBF2*. (b) Comparison of FA composition in seeds of three independent *Ph‐GUS* and *Ph‐DPBF2* T2 generation transgenic plants. FA content represents the mean (±SE) from three independent biological replicates. (c) Comparison of *DPBF2* and FA biosynthesis gene expression in S6 and S7 stage of developing siliques between *Ph‐GUS* and *Ph‐DPBF2* transformed plants. RT‐qPCR analysis of *DPBF2*, *FAD2*, *FAD3*, *LPCAT1/2*, *PDCT*, and *FAE1* in the *Ph‐GUS* and *Ph*‐*DPBF2* overexpression lines containing the phaseolin promoter. The S6 and S7 siliques used in this study included seeds of the walking‐stick embryo and curled cotyledon phase, respectively. Statistically significant differences by unpaired *t* test are indicated (*p < .05, **p < .01, ***p < .001). Relative expression values are given in comparison with the WT (WT = 1). Mean (±SE) from three independent biological replicates

To investigate the role of DPBF2 in seed, *DPBF2* was overexpressed during seed development under the control of seed‐specific phaseolin promoter (Figure 6). Transgenic plants heterologously expressing the *GUS* gene from the phaseolin promoter were used as controls (Figure 6a). T3 seed FA analysis was performed on three T2 independent lines of *Ph‐GUS* or *Ph‐DPBF2*. As a result, seed‐specific *DPBF2* overexpression increased 18:2 and 20:1 and decreased 18:1 and 18:3 compared with the control *GUS* overexpression (Figure 6b). The result was a change in FA composition similar to the *dpbf2‐1* knock‐out mutant (Figure 4a). In addition, seed FA composition of *dpbf2‐1* transformed with *Ph‐DPBF2* was analyzed (Figure S4). FA composition of T3 seed harvested from three independent T2 transgenic lines showed the same change in FA composition as *dpbf2‐1* (Figure 4a) and transgenics overexpressing *Ph‐DBPF2* (WT + *Ph‐DBPF2*) (Figure 6b).

To find the cause of the change in the composition of seed FAs in WT + *Ph‐DBPF2* lines, the expression of unsaturated FA synthesis‐related genes was analyzed in S6 siliques containing walking‐stick embryo stages and S7 stage siliques containing curled cotyledon stages in *Ph‐DPBF2 #2* line and *Ph‐GUS #1* control. *Ph‐DPBF2 #2* line, showed a 7.2‐fold in S6 and 8.1‐fold in S7 increase in *DPBF2* expression compared with *Ph‐GUS #1* line (Figure 6c). Although *DPBF2* increased very subtly upon transition from S6 to S7, the expression of *FAD2*, *FAD3*, *LPCAT1*, *LPCAT2*, *PDCT*, and *FAE1* decreased from S6 to S7 stage in the *Ph‐GUS #1* line. In contrast, in *Ph‐DPBF2 #2*, these genes were upregulated in the S6 stage and remained high through the S7 stage (Figure 6c). These results indicated that during seed development, seed‐specific overexpression of *DPBF2* increased *FAD2*, *FAD3*, *LPCAT1*, and *LPCAT2* slightly and *PDCT* and *FAE1* highly. Taken together, increased expression of *DPBF2* during different stages of seed development can affect the regulation of many FA synthesis genes and contribute to changes in the unsaturated FA composition of seed TAG.

### 3.7 DPBF2/L1L/NF‐YC2 complex upregulates PDCT and FAE1 expression

**FIGURE 7:**
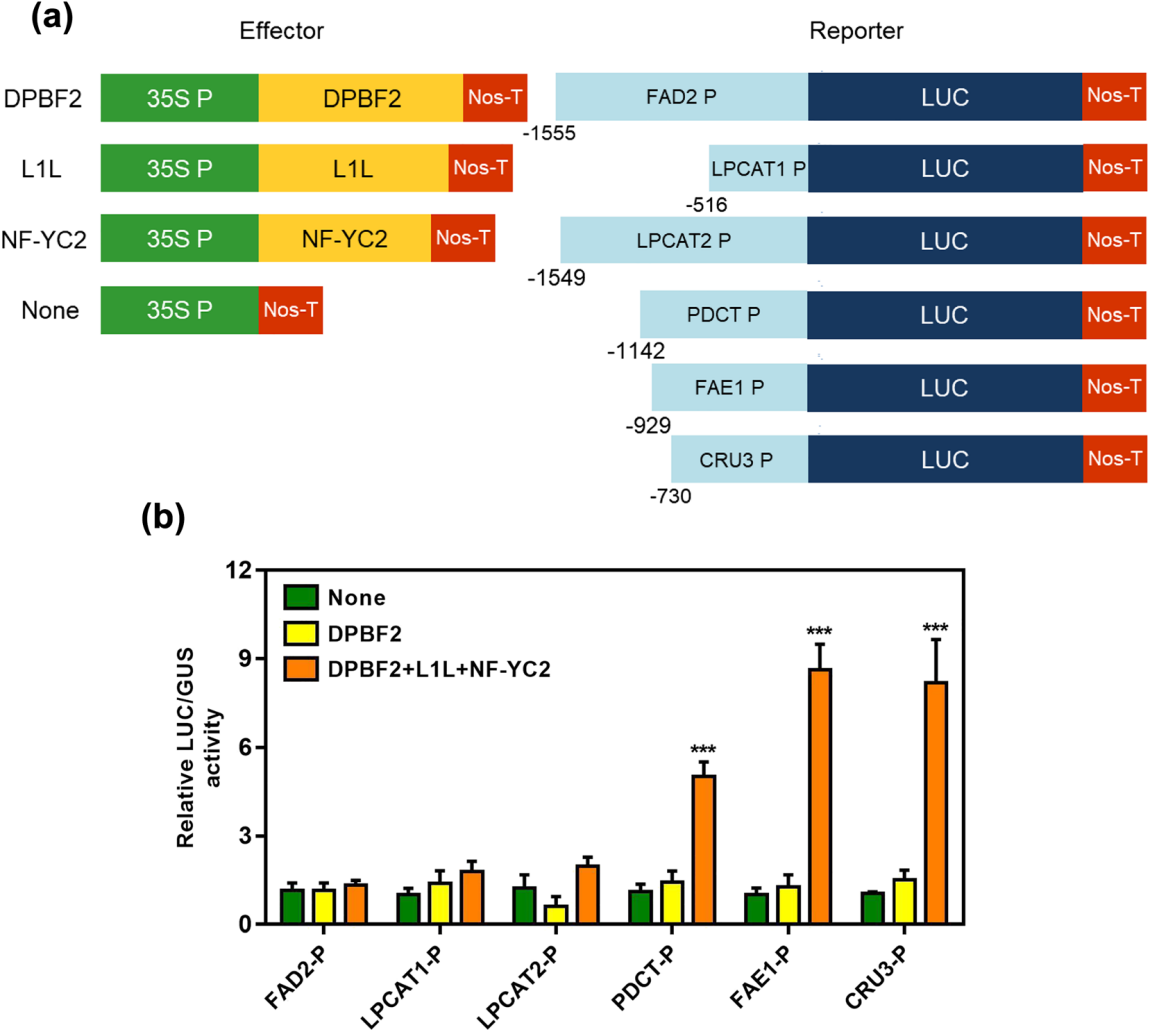
FA biosynthesis genes transcriptional activation assay by DPBF2 and DPBF2/L1L/NF‐YC2 complex in *Nicotiana benthamiana* leaf protoplasts. (a) Schematic diagrams of reporter and effector constructs. In the effector construct, DPBF2, L1L, and NF‐YC2 were cloned between the CaMV 35S promoter and the terminator of the nopaline synthase gene (Nos‐T). In the reporter constructs, the promoter of FA biosynthesis genes was fused to the luciferase gene. (b) Transcriptional activation assay in *Nicotiana benthamiana* leaf protoplasts. The effector and reporter constructs shown in (a) were cotransfected into protoplasts, and luciferase activities were determined fluorometrically. *GUS* gene expression was used to normalize the luciferase activities, and four measurements were averaged (*t* test, ***p < .001). The bars indicate the SEM

In previous reports, it has been identified that DPBF2 regulates seed storage protein and FA biosynthesis genes (*CRU3* and *FAD3*) with LEC1‐LIKE (L1L), NF‐YC2 by binding G‐Box ACGT core sequence (Mendes et al., 2013; Yamamoto et al., 2009). Therefore, we performed a transcriptional activity assay with DPBF2, L1L, and NF‐YC2 transient coexpression to explain how DPBF2 can regulate FA biosynthesis genes (Figure 7a). The *FAD2* and *LPCAT1/2* promoter were not activated by expression of DPBF2 alone and DPBF2 with L1L and NF‐YC2 (Figure 7b). But *PDCT* and *FAE1* promoters exhibited 5‐ and 8.6‐fold activation by coexpression of DPBF2 with L1L and NF‐YC2 compared with control (None), nor by DPBF2 alone (Figure 7b). The *CRU3* promoter used for positive control was activated 8.1‐fold compared with control, which is consistent with results in Yamamoto et al. (2009). The promoter in all FA synthesis genes and *CRU3* was included at least three or more putative DPBF2‐binding motifs (ACGT) although *FAD2* and *LPCAT1/2* had not changed in promoter activation (Figure S5). These results reveal that DBPF2 regulates *PDCT* and *FAE1* together with L1L and NF‐YC2 to control FA composition.

## 4 DISCUSSION

TAG, which accumulates during seed development, mediates the storage of a large amount of unsaturated FAs in Arabidopsis seeds. In this study, we report that DPBF2 transcription factor regulates the FA composition in the TAG of seeds. Spatial–temporal expression of *DPBF2* was seed specific and started during cotyledon and axis development in seeds (Figure 1c,d). This suggests that *DPBF2* is a seed‐specific gene and related to genes involved in FA modification. *DPBF2/bZIP67* has the highest expression level in siliques at 10–13 days after pollination (DAP) (Bensmihen et al., 2002), and transgenic Arabidopsis embryos carrying bZIP67 promoter:GFP‐tagged *bZIP67* exhibit fluorescence from 8 DAP to 13DAP (Bensmihen et al., 2005). In addition, the eFP browser (http://bar.utoronto.ca/efp2/Arabidopsis/Arabidopsis_eFPBrowser2.html) describes locus AT3G44460 for *DPBF2* as being seed‐specific and most strongly expressed in the middle stages of seed development (Winter et al., 2007).

*DPBF2* is regulated by the master regulator LEC2. *DPBF2* expression began at the embryo stage when *LEC2* expression reached its peak (Figure 1d). In addition, the expression of *DPBF2* was decreased in developing seeds of *lec2‐1* mutant (Figure 1b). *LEC2* expression significantly increased *DPBF2* expression in transgenic lines (Figure 1a) and protoplasts (Figure 2). Mutation of the RY motif region present in the *DPBF2* promoter reduced the transcriptional activation of *DPBF2* by LEC2, and this result showed that LEC2 directly regulated the expression of *DPBF2* (Figure 2).

The demonstration that DPBF2 was targeted to the nucleus and had transcriptional activity when transgenically expressed in yeast supports the notion that DPBF2 is a transcription factor (Figure 3). Transformation of DPBF2 fused with the GAL4 DB domain into yeast harboring GAL4‐binding sites fused to a *lacZ* gene produces strong β‐galactosidase activity, indicating that DPBF2 has transcriptional activity in yeast (Kim et al., 2002). bZIP67 is localized to the nucleus in Arabidopsis containing bZIP67 promoter:GFP‐tagged *bZIP67* (Bensmihen et al., 2005). In vitro and in vivo experiments have confirmed that DPBF2/bZIP67 binds to the *FAD3* promoter region (Mendes et al., 2013).

The *dpbf2‐1* mutant used in this study represents the same T‐DNA inserted mutant to *bzip67‐1* in the same *DPBF2*/*bZIP67* gene. In *dpbf2‐1*/*bzip67‐1* (Salk_085497C), a T‐DNA is located in the second intron (Figure S1), and in *bzip67‐2* (GABI314D04), the T‐DNA is inserted into the third intron (Mendes et al., 2013). In *dpbf2‐1*, the proportion of 18:1 and 18:3 FAs decreased and that of 18:2 FA increased compared with those in the WT (Figure 4a). This is slightly different from the seed FA composition of *bzip67‐1*, but the tendency of each FA to increase and decrease is consistent (Mendes et al., 2013). Seed FA content of *dpbf2‐1* was about 93% of the WT (Figure 4b). Similarly, the total FA content, protein content, and seed weight of *bzip67‐1* seeds did not change compared with those of WT (Mendes et al., 2013). This showed that, unlike LEC2, DPBF2 did not have a critical effect on seed oil content but was a transcription factor that affects FA composition. Mendes et al. (2013) reported that DPBF2/bZIP67 regulates the transcription of *FAD3*. We also observed downregulation of *FAD3* in developing seeds of the *dpbf2‐1* mutant (Figure 4c).

DPBF2 exerted a dose‐dependent effect on the FA composition (Figure 5). The proportion of 18:1 and 18:3 FAs are decreased and 18:2 is increased in *bzip67‐1*. In bZIP67 overexpressors, 18:1 and 18:2 are increased and 18:3 is decreased compared with the WT (Mendes et al., 2013). Changes in FA composition of *dpb2‐1* mutant seeds are the same as reported by Mendes et al. (2013). The increase of 18:2 and the decrease of 18:3 in the *DPBF2* overexpressing seeds produced in this study were the same as those reported by Mendes et al. (2013), but the change of 18:1 was different. Such a subtle difference is likely due to the difference in expression timing and amount of DPBF2/bZIP67 by phaseolin or glycine seed‐specific promoters.

Compared with *bzip67‐1*, the expression level of *FAD3* in *dpbf2‐1* was reduced to a lesser extent than in the WT, but that the expression of *PDCT/ROD1* was similarly increased compared with that of the WT (Figure 4c). Mendes et al. (2013) suggested that the decreased *FAD3* transcript levels and increased *PDCT* transcript levels in developing stage 8 siliques of *bzip67‐1* result in a decrease in 18:3 and 18:1 FAs content. We showed that transcript levels of *LPCAT1* and *LPCAT2* dropped to 68% relative to those of the WT in *dpbf2‐1* (Figure 4c). *lpcat1*/*2* show a slight decrease in 16:0 and 18:1 FA contents and a 2% decrease in both 18:2 and 18:3 FAs compared with those of WT (Bates et al., 2012; Wang et al., 2012), whereas C_20–22_ unsaturated FA contents are increased to 33.5% compared with 26.5% in the WT (Bates et al., 2012). The increase in C_20–22_ unsaturated FA content is most likely due to the increase in C_20_ unsaturated fatty acyl‐CoA, a substrate that can naturally acylate to TAG. Based on the above report, a slight increase of 20:1 in *dpbf2‐1* mutant is likely due to decreased expression of *LPCAT1* and *LPCAT2* (Figure 4a,c).

In the Arabidopsis *fad3* mutant, the 18:3 FA content is decreased, and the 18:1 and 18:2 FA contents are increased (James & Dooner, 1990; Lemieux et al., 1990). The expression levels of *FAD2* were upregulated in *dpbf2‐1*, so the seed FA composition of *dpbf2‐1* is likely to be a mix of the FA compositions of *fad3* mutant and *FAD2* overexpressor (Figure 4). Therefore, in slight contrast to the FA composition of *fad3*, the decrease of 18:1 in *dpbf2‐1* seed may be due to downregulation of *FAD3* and upregulation of *FAD2* in the *dpbf2‐1* knock‐out mutant.

Seed‐specific overexpression of *DPBF2* in WT and *dpbf2‐1* mutants showed a very similar phenotype to the FA composition of *dpbf2‐1* knock‐out mutant seeds (Figures 4a, 6b, and S4). 18:1 and 18:3 decreased while 18:2 and 20:1 increased (Figures 4a, 6b, and S4). This may be due to that DPBF2 upregulated a number of genes related to the synthesis of unsaturated FAs, which ultimately control FA composition regardless of excessive *DPBF2* expression during seed development.

Transcriptional activation assay was performed on the *FAD2*, *FAD3*, *LPCAT1*, *LPCAT2*, *PDCT*, and *FAE1* genes, which showed changes in expression in *dpbf2‐1* and *Ph‐DPBF2* overexpressors (Figure 4c, 6c, and 7). As a result, *PDCT* and *FAE1* expression was upregulated when DPBF2 was combined with L1L and NF‐YC2 rather than alone (Figure 7). We predicted DPBF2‐binding motif (ACGT) in the promoter region of *PDCT* and *FAE1* genes, there were at least seven motifs in both of forward and reverse sequence (Figure S5). We speculate that DPBF2 may regulate *PDCT* and *FAE1* transactivation by binding these sites. But it should be confirmed which position will be bound with DPBF2, in effect. Although *FAD2*, *LPCAT1*, and *LPCAT2* expression showed no changes in transactivation, it is possible that DPBF2 regulates these genes with other transcription factors because it has been reported that DPBF2 functions with various transcription factors (Bryant et al., 2019; Jo et al., 2020; Yamamoto et al., 2009). Taken together, these results suggest that DPBF2 works together with other transcription factors, such as L1L and NF‐YC2 to regulate the synthesis of unsaturated fatty acids in TAG during seed development.

**FIGURE 8:**
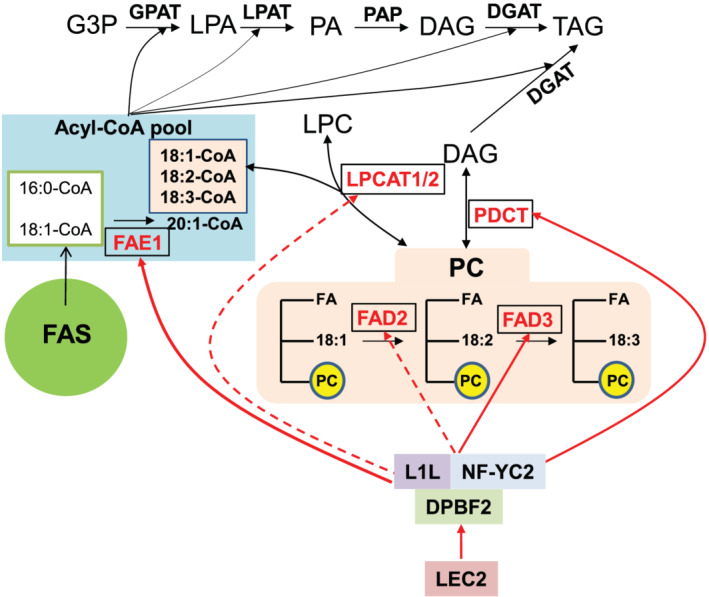
Model for the LEC2/DPBF2 network‐mediated regulation of polyunsaturated fatty acid biosynthesis and accumulation in triacylglycerol (TAG) in Arabidopsis seeds. DPBF2 regulates *FAD2*, *FAD3*, *LPCAT1*, *LPCAT2*, *PDCT*, and *FAE1* expression. Black solid arrows represent metabolic reactions. Red solid arrows represent positive control by DPBF2/L1L/NF‐YC2 complex. Red dotted arrows represent possible positive control by DPBF2 together with other unknown factors. G3P: glycerol 3‐phosphate; LPA: lysophosphatidic acid; PA: phosphatidic acid; DAG: diacylglycerol; TAG: triacylglycerol; LPC: lysophosphatidylcholine; PC: phosphatidylcholine; GPAT: glycerol‐3‐phosphate acyltransferase; LPAT: lysophosphatidic acid acyltransferase; PAP: phosphatidic acid phosphatase; DGAT: diacylglycerol acyltransferase; FAS: fatty acid synthase; FAE1: fatty acid elongase 1; LPCAT1/2: lysophosphatidylcholine acyltransferase 1/2; PDCT: phospholipid:diacylglycerol cholinephosphotransferase; FAD2: fatty acid desaturase 2; FAD3: fatty acid desaturase 3

In conclusion, *DPBF2* is a seed‐specific transcription factor regulated directly by LEC2 that controls the expression of genes regulating the degree of unsaturation of seed FAs, such as 18:1, 18:2, 18:3, and 20:1, in TAG accumulation (described in Figure 8). Our results demonstrated that DBPF2 with L1L and NF‐YC2 positively controls the expression of *PDCT* and *FAE1* together with the previously reported *FAD3*. However, it remains to be determined whether DPBF2 directly or indirectly modulates *FAD2*, *LPCAT1*, and *LPCAT2* genes in combination with various transcription factors.

## CONFLICT OF INTEREST

The authors declare no conflicts of interest.

## AUTHOR CONTRIBUTIONS

HUK, IK, and KRL performed the experiments and wrote the paper. MEP performed fatty acid analysis. All authors read and approved the final manuscript.

## Supporting information


**Table S1.** Primers used for this study.
**Table S2.** Identities of transcription factors expressed at higher levels in senescing leaves at the S1 (100% green leaves, 30 days after germination) or S3 (50% yellow leaves) stages of the OIL21 line, which expresses senescence‐induced LEC2, than in wild‐type senescing leaves at the corresponding stages.

**Figure S1.** Identification of a *dpbf2‐1* T‐DNA insertion knock‐out mutant.
**Figure S2.** Leaf fatty acid content analysis in WT and *dpbf2‐1* line.
**Figure S3.** Overexpression of *35S:DPBF2* in WT plants.
**Figure S4.** Seed fatty acid composition of WT and three independent *dpbf2‐1+Ph‐DPBF2* T2 generation transgenic plants.
**Figure S5.** The *cis*‐element in the promoter region of six genes.

## Data Availability

The data performed in this study are presented in the paper and the Supporting Information.
