# Supplementary material for: The seed‐specific transcription factor DPBF2 modulates the fatty acid composition in seeds
Source: Plant Direct. 2022 Apr 3;6(4):e395. doi: 10.1002/pld3.395 (PMC8977579; doi:10.1002/pld3.395)
Supplement: Supplementary file 2 — Figure S1. Identification of a dpbf2‐1 T‐DNA insertion knock‐out mutant. Figure S2. Leaf fatty acid content analysis in WT and dpbf2‐1 line. Figure S3. Overexpression of 35S:DPBF2 in WT plants. Figure S4. Seed fatty acid composition of WT and three independent dpbf2‐1+Ph‐DPBF2 T2 generation transgenic plants. Figure S5. The cis‐element in the promoter region of six genes. [file PLD3-6-e395-s001.pptx]

## Slide 1
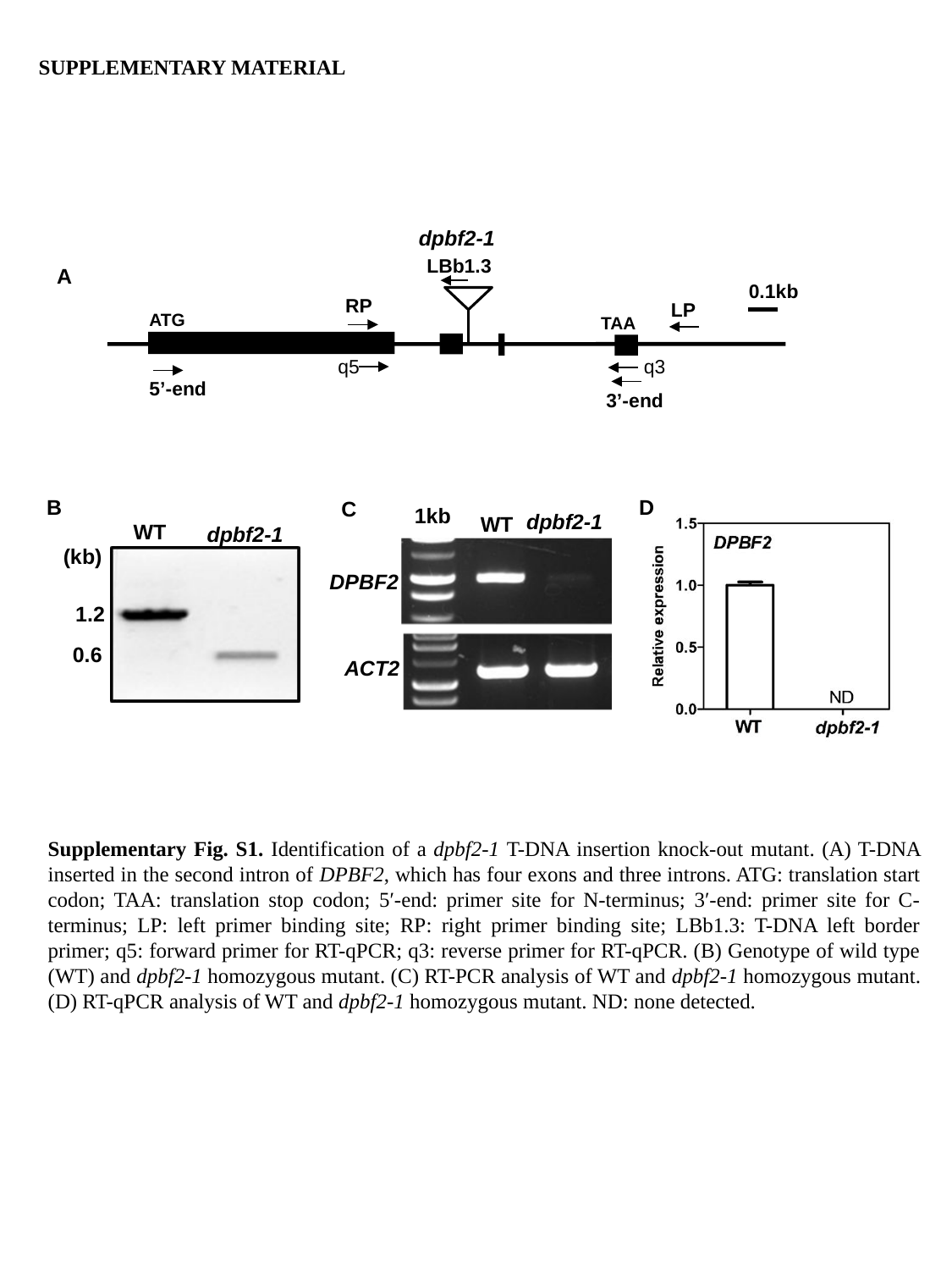

SUPPLEMENTARY MATERIAL
dpbf2-1
LBb1.3
A
0.1kb
RP
LP
ATG
TAA
q3
q5
5’-end
3’-end
B
D
C
1kb
dpbf2-1
WT
DPBF2
ACT2
WT
dpbf2-1
1.2
(kb)
0.6
Supplementary Fig. S1. Identification of a dpbf2-1 T-DNA insertion knock-out mutant. (A) T-DNA inserted in the second intron of DPBF2, which has four exons and three introns. ATG: translation start codon; TAA: translation stop codon; 5′-end: primer site for N-terminus; 3′-end: primer site for C-terminus; LP: left primer binding site; RP: right primer binding site; LBb1.3: T-DNA left border primer; q5: forward primer for RT-qPCR; q3: reverse primer for RT-qPCR. (B) Genotype of wild type (WT) and dpbf2-1 homozygous mutant. (C) RT-PCR analysis of WT and dpbf2-1 homozygous mutant. (D) RT-qPCR analysis of WT and dpbf2-1 homozygous mutant. ND: none detected.

## Slide 2
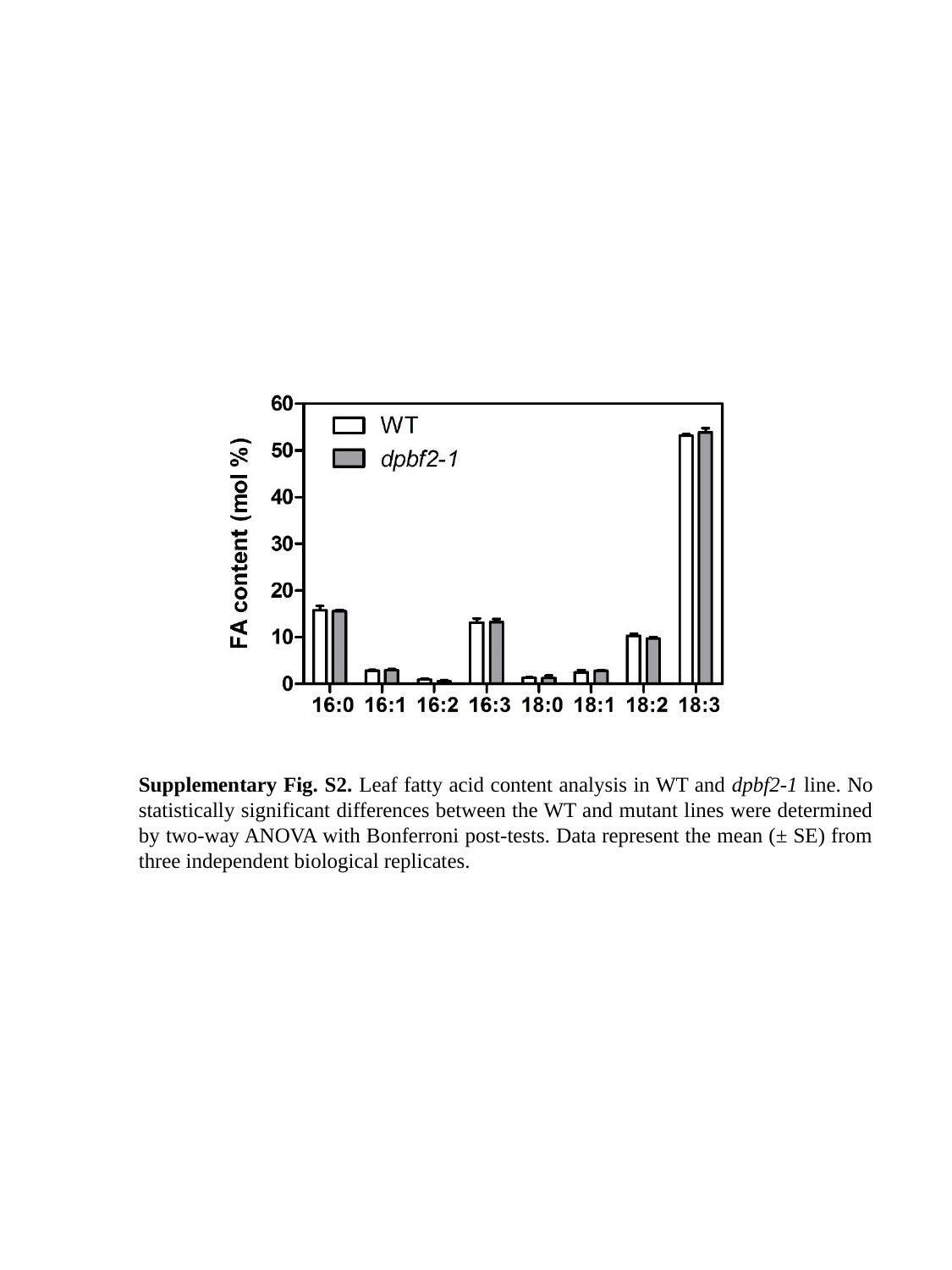

Supplementary Fig. S2. Leaf fatty acid content analysis in WT and dpbf2-1 line. No statistically significant differences between the WT and mutant lines were determined by two-way ANOVA with Bonferroni post-tests. Data represent the mean (± SE) from three independent biological replicates.

## Slide 3
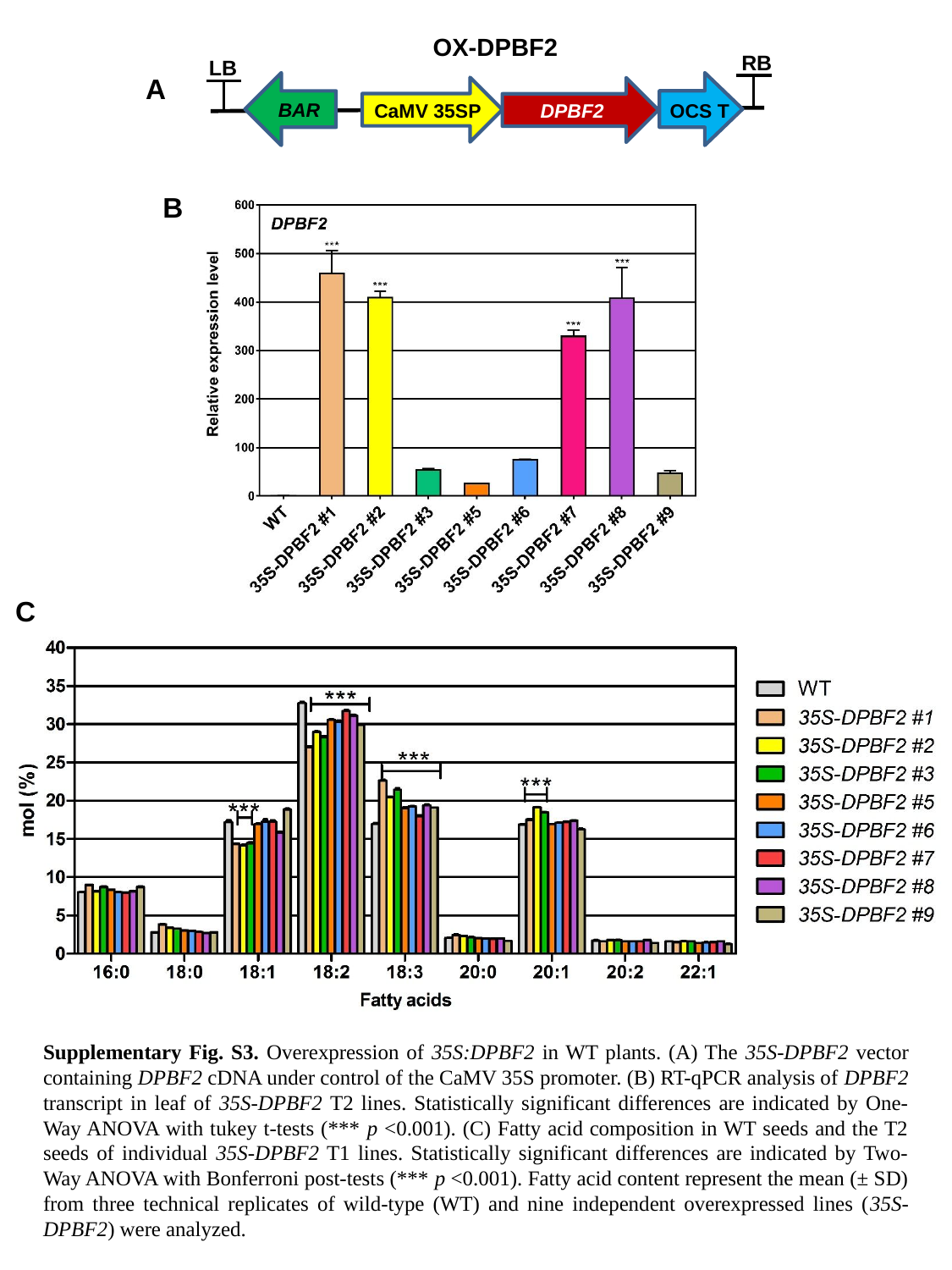

OX-DPBF2
RB
LB
DPBF2
 BAR
CaMV 35SP
 OCS T
A
B
C
Supplementary Fig. S3. Overexpression of 35S:DPBF2 in WT plants. (A) The 35S-DPBF2 vector containing DPBF2 cDNA under control of the CaMV 35S promoter. (B) RT-qPCR analysis of DPBF2 transcript in leaf of 35S-DPBF2 T2 lines. Statistically significant differences are indicated by One-Way ANOVA with tukey t-tests (*** p <0.001). (C) Fatty acid composition in WT seeds and the T2 seeds of individual 35S-DPBF2 T1 lines. Statistically significant differences are indicated by Two-Way ANOVA with Bonferroni post-tests (*** p <0.001). Fatty acid content represent the mean (± SD) from three technical replicates of wild-type (WT) and nine independent overexpressed lines (35S-DPBF2) were analyzed.

## Slide 4
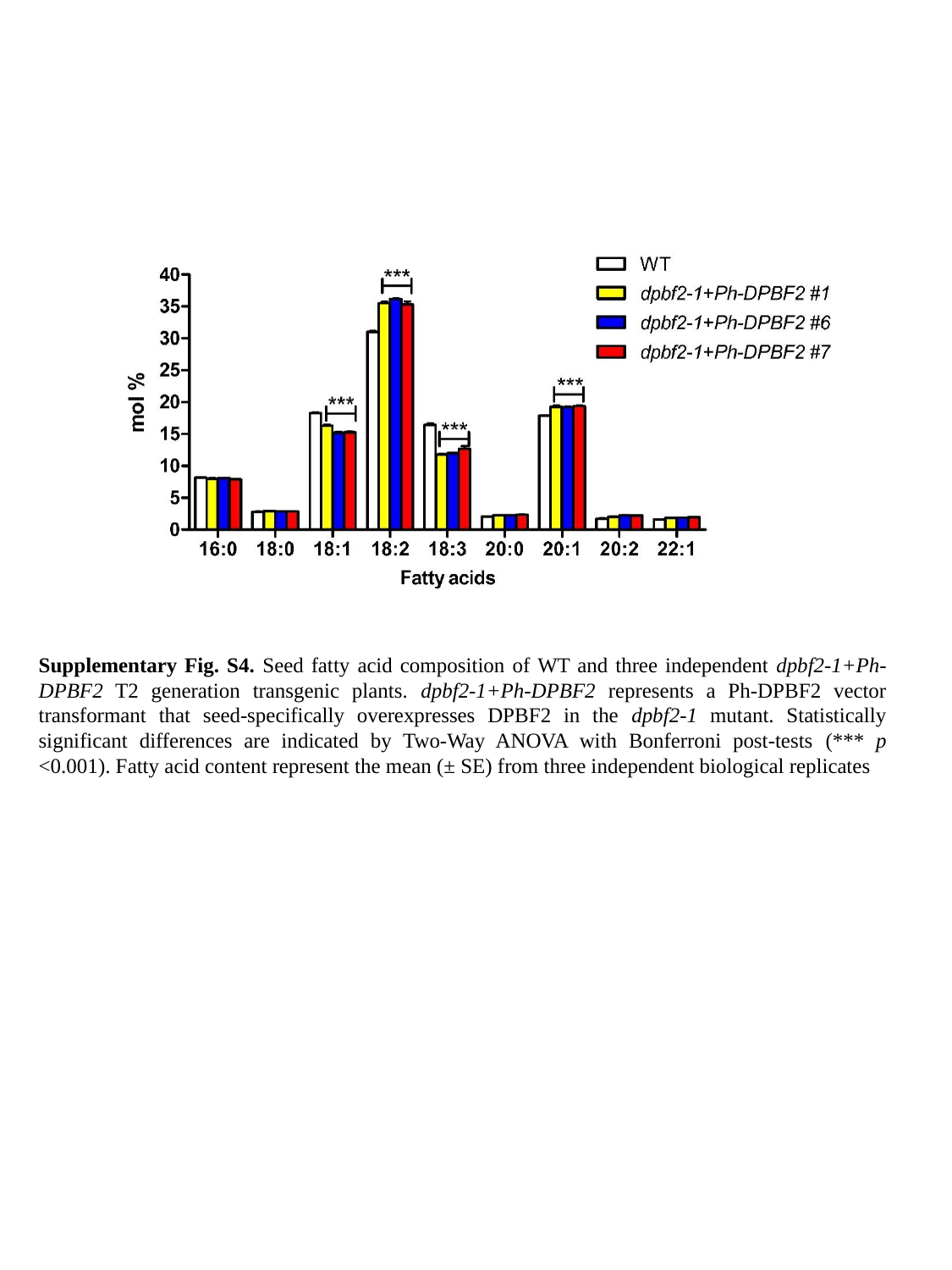

Supplementary Fig. S4. Seed fatty acid composition of WT and three independent dpbf2-1+Ph-DPBF2 T2 generation transgenic plants. dpbf2-1+Ph-DPBF2 represents a Ph-DPBF2 vector transformant that seed-specifically overexpresses DPBF2 in the dpbf2-1 mutant. Statistically significant differences are indicated by Two-Way ANOVA with Bonferroni post-tests (*** p <0.001). Fatty acid content represent the mean (± SE) from three independent biological replicates

## Slide 5
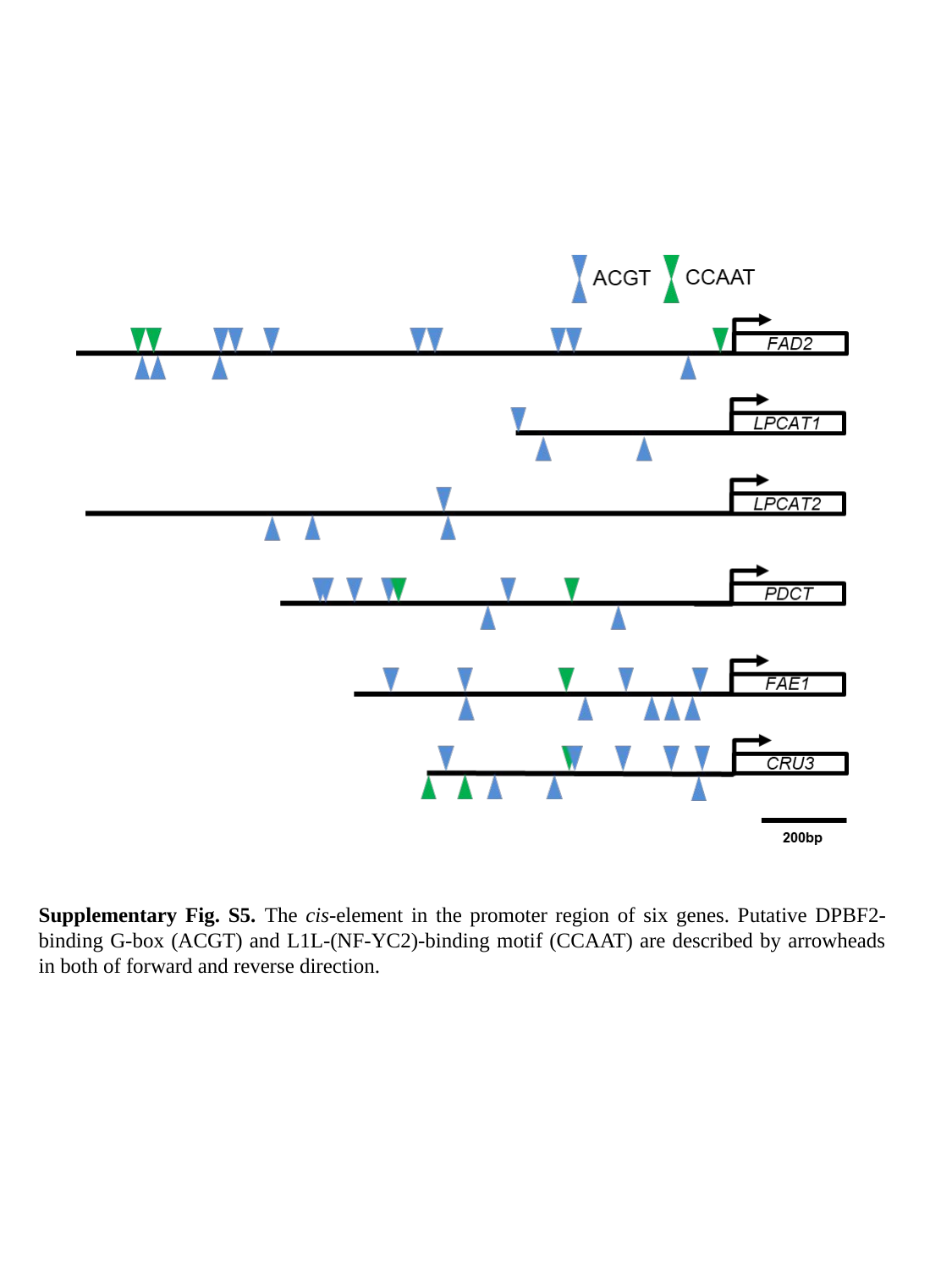

Supplementary Fig. S5. The cis-element in the promoter region of six genes. Putative DPBF2-binding G-box (ACGT) and L1L-(NF-YC2)-binding motif (CCAAT) are described by arrowheads in both of forward and reverse direction.
